# Non-communicable diseases in antiretroviral therapy recipients in Kagera Tanzania: a cross-sectional study

**DOI:** 10.11604/pamj.2013.16.84.2831

**Published:** 2013-11-06

**Authors:** Mgaywa Gilbert Mjungu Damas Magafu, Kazuhiko Moji, Ehimario Uche Igumbor, Naoko Shimizu Magafu, Michael Mwandri, Julius Chacha Mwita, Dereje Habte, Godfrey Mutashambara Rwegerera, Masahiro Hashizume

**Affiliations:** 1 University of Botswana, Gaborone, Botswana; 2 Research Institute for Humanity and Nature, Kyoto, Japan; 3 School of Public Health, University of the Western Cape, Bellville, South Africa; 4 Japan Overseas Christian Medical Co-operative Services, C/o Roman Catholic Archdiocese of Tabora, Private Bag, Tabora, Tanzania; 5 Institute of Tropical Medicine, University of Nagasaki, Nagasaki, Japan

**Keywords:** Non-communicable diseases, HIV/AIDS, highly active antiretroviral therapy, health-related quality of life, Tanzania

## Abstract

**Introduction:**

The aim of this study was to describe the extent of self-reported non-communicable diseases (NCDs) among highly active antiretroviral therapy (HAART) recipients in Kagera region in Tanzania and their effect on health-related quality of life (HRQOL). This study was conducted 2 years after HAART administration was started in Kagera region.

**Methods:**

The SF-36 questionnaire was used to collect the HRQOL data of 329 HAART recipients. Questions on the NCDs, socio-demographic characteristics and treatment information were validated and added to the SF-36. Bivariate analyses involving socio-demographic characteristics and SF-36 scores of the recipients were performed. Multiple logistic regression was employed to compute adjusted odds ratios for different explanatory variables on physical functioning and mental health scores.

**Results:**

Respondents who reported having 1 or more NCDs were 57.8% of all the respondents. Arthritis was the commonest NCD (57.8%). Respondents with the NCDs were more likely to have HRQOL scores below the mean of the general Tanzanian population. The population attributable fraction (PAF) for the NCDs on physical functioning was 0.28 and on mental health was 0.22.

**Conclusion:**

Self-reported NCDs were prevalent among the HAART recipients in Kagera region. They accounted for 28% of the physical functioning scores and 22% of the mental health scores that were below the mean of the general Tanzanian population. Therefore, the integration of NCD care is important in the management of HIV/AIDS.

## Introduction

In Tanzania, the administration of highly active antiretroviral therapy (HAART) to eligible recipients started in 2004 with the establishment of HIV/AIDS care and treatment centres (CTCs) in selected regions [1]. The aim of the HIV/AIDS care and treatment programme is to provide comprehensive and quality, continuing care and treatment to eligible recipients in order to improve their longevity and health-related quality of life (HRQOL) [2]. Kagera Regional Hospital started administering HAART in 2005 [3]. A study conducted 2 years after HAART administration was commenced at the hospital showed that the HRQOL of the HAART recipients was comparable in some aspects to that of the general Tanzanian population [4].

Although antiretroviral drugs (ARVs) do not cure HIV/AIDS, they have incredibly reduced the rates of mortality and morbidity, improved the quality of life, revitalised communities and transformed the perception of HIV/AIDS from a deadly disease to a manageable chronic illness [5–10]. The life expectancy of HAART recipients has increased [5, 11], thus, like any other aging population, the recipients begin to suffer from an increased occurrence of chronic diseases including NCDs [11]. This phenomenon may represent a sort of an epidemiological transition whereby infectious diseases overlap with NCDs. Inflammation by HIV, HAART effect, opportunistic infections or their treatment, and traditional risk factors like obesity are among the factors which have been cited for the high prevalence of NCDs in people living with HIV/AIDS (PLWHA) [6, 7, 12]. Although the prevalence of NCDs in sub-Saharan Africa is high and is projected to continue increasing [13, 14], the prevalence and trends of NCDs in the general population and PLWHA in Kagera region had not been studied at the time of this study. The aim of this study was to describe self-reported NCDs among HAART recipients in Kagera and to examine how the diseases affect the recipients’ HRQOL. Results from this study may guide health policy-makers and clinicians in making decisions on how to best manage both HIV/AIDS and the NCDs.

## Methods

### Study design, setting and population

This was a cross-sectional study comprising HAART recipients aged 18 years and above at the CTC of the Kagera Regional Hospital in Tanzania conducted in 2007. The hospital is situated in Bukoba town in north-western Tanzania [3]. All study participants had been on HAART for at least 6 months.

### Sample size computation

At data collection, the CTC had 1,521 HAART recipients. After applying the exclusion criteria of the study (age less than 18 years, time on therapy less than 6 months and pregnancy), 892 recipients remained and served as the sampling frame. The sample size was calculated using StatCalc within Epi Info version 3.4.3 (CDC, Atlanta, GA, USA). The expected frequency of the factor under study, namely the prevalence of NCDs, was unknown thus was assumed to be 50%, which gave the largest sample. The sample size was calculated in such a way that the expected frequency of NCDs lay within 5 points of the presumed frequency, that is, 50±5%. Therefore, the worst acceptable result was set within the range 45-55%. At 95% confidence interval (CI), the sample size was calculated to be 269. However, in anticipation of some non-response, 22% of the calculated sample size (60 participants) was added giving a total sample size of 329 recipients. None of the study HAART recipients selected to participate in this study refused.

### Sampling procedure

Simple random sampling method was employed. First a unique number was assigned to each of the HAART recipients in the sampling frame. A table of random numbers was used to randomly select the HAART recipients, one by one from a sampling frame of 892, until the required sample size (329) was obtained. Trained interviewers met the randomly selected HAART recipients when they attended the CTC and requested them to participate in the study. After obtaining their informed consent, the selected recipients were interviewed.

### Data collection and instrument

Data on HRQOL were collected in face-to-face interviews using the Swahili version of the SF-36 questionnaire. The SF-36 has internal consistency and strong psychometric properties and has been validated for use in more than 40 languages [15, 16] including Swahili, the national language of Tanzania [15]. It has 8 multi-item scales that assess respondents’ physical functioning, role limitation due to physical health problems, bodily pain, general health perceptions, vitality, social functioning, role limitation due to emotional health problems and mental health [17]. Apart from the 8 scales of the SF-36, questions on socio-demographic information, NCDs or their sequelae like cerebro-vascular accidents, self-reported adherence to ARVs and perceived untoward effects of HAART were added to the questionnaire. An NCD was defined as a disease or medical condition that was not transmissible among people (i.e. non-infectious) [14] that had been diagnosed by a medical doctor in the past. All chronic but infectious diseases were excluded. Peptic ulcers were included because their causation was considered to be multifactorial. The NCDs were self-reported. Kagera Regional Hospital was the only place where HAART services were available for the whole region at the time of the study. Patients’ NCD records were kept at the different hospitals and health centres, scattered in the region, where the patients came from. Thus, it was practically impossible to confirm reported NCDs by checking the records. The question on NCDs consisted of a list of well-known NCDs and an "other, specify" option for NCDs that were not on the list.

Adherence was recorded, and later in the analysis stage categorised, as perfect when recipients reported that they had taken 100% of all the prescribed ARV doses in the past 1 week before they were interviewed and near-perfect when ≥95% but <100% of all prescribed doses were reported to have been taken [18]. The remainder were classified as non-adherent. For the perceived untoward effects of HAART, recipients were asked whether they have had bad symptoms or conditions in the past one month thought to be caused by HAART. A pilot study was conducted to validate the questions on the presence or absence of NCDs and the other questions that were added to the SF-36. Treatment information, the first and the latest available CD4+ cell counts were retrieved from the patients’ records. The time duration between the 2 cell counts was also recorded. A pilot study was conducted before data collection to validate the questions that were added to the SF-36.

### Ethical considerations

This study was first approved by the Medical Research Coordinating Committee (MRCC) of the National Institute for Medical Research in Tanzania. Evidence of MRCC's ethical clearance was then presented to the Kagera Region Health Services. In the field, the nature of the study and other items in the informed consent form and the participant information sheet were explained to the prospective study participants by the interviewers. Participants were asked to sign (or thumbprint) the consent form when it was established that they had understood the nature of the research and had given their consent voluntarily.

### Data handling and cleaning

Data were entered separately in MS Excel spreadsheet by 2 people. The 2 datasets were compared and discrepancies were corrected. The data were then imported into Stata SE 11 for Windows (StataCorp, College Station, TX, USA) and the data were checked and cleaned using codebook and frequency tabulations.

### Scoring of the SF-36

The SF-36 item scales should be scored so that the higher the score the better the health state [19]. To meet this criterion, items of the SF-36 that required recoding were recalibrated. After item recoding, scale scores were computed by summing across items in the same scale to give raw scale scores. The score was a simple algebraic sum of the responses for all the items in the scale [19]. In the next step, the raw scores were transformed into a 0-100 scale that was used in the analysis.

### Variable categorisation

Dichotomous variables were sex (male/female), religion (Christian/Muslim), NCDs (yes/no), reported side effects of HAART (yes/no), perfect or near-perfect adherence to ARVs (yes/no) and CD4+ cell increase over time (yes/no). The CD4+ cell change over time was derived from the difference between the last CD4+ cell measurement and the first one divided by the time span in months between the 2 counts. The results fell into 3 categories: decrease, no change and increase. An increase was classified as "yes" and no change or decrease was classified as "no" for the variable. The other variables namely marital status, level of education, income level per month and employment had more than 2 categories. The 8 scale scores of the SF-36 questionnaire were also dichotomised. The cut-off point for dichotomisation for each of the scale scores was the respective mean scale scores of the general Tanzanian population [15]. Those who scored under the mean for the respective scale for the general population were categorised as "below" and those who scored equal to or above the mean of the general population were classified as "above".

### Analysis

Frequency tables for categorical variables like sex and summary statistics for quantitative variables like age were made. Bivariate and multiple logistic regression analyses were later performed. The explanatory variables entered in the logistic regression equation were those which were stated in the literature to be associated with the HRQOL of the HAART recipients [20–24] or those which were statistically significant in the bivariate analysis. The variables were NCDs, CD4+ cell count change, HAART adverse effects, adherence to ARVs, gender, age, educational level and income level per month. The outcome variables, tested separately, were physical functioning and mental health. Physical functioning scale scores represented the physical health of the HAART recipients while mental health scale scores represented their mental health.

Categories for the explanatory variables were as follows: NCDs (yes/no), HAART side effects (yes/no), adherence to ARVs (perfect or near-perfect/not), CD4+ cell change (yes/no), sex (male/female), age-group in years (≤24, 25-34, 35-44, 45-54, 55-64 or ≥65), education level of the respondent (no formal education, primary education or post-primary education) and income level per month in Tanzania shillings (TZS) (<100,000; 100,000-300,000; or >300,000). Except for age-group, education level and income per month, all the variables entered in the equations were dichotomous. The non-dichotomous variables were entered in the model in the form of indicator contrasts. The number of variables entered in the model was less than 10% of the events (number of participants scoring less than the mean for the general population), which is usually recommended [25]. Before running the backward stepwise procedure that was used to select the variables to be included in the final model, collinearity of the variables was tested and found to be acceptable. The logistic regression procedure was based on the global likelihood ratio test. The significance level for variable entry into the model was set at 0.05 and that for removal from the model was set at 0.10. The Hosmer-Lemeshow test was employed to check the goodness-of-fit of the model [26]. The population attributable fraction (PAF) for the NCDs, which represents the item scale scores that were below the mean of the general population as a result of the presence of the NCDs, was computed directly using the statistical software used for this analysis i.e. STATA SE 11 for Windows.

## Results

### Sample characteristics

The study comprised 329 participants (Table 1). The mean (standard deviation (SD)) age of the study participants was 40.9 (8.7) years. Females (n = 217; 66.0%) made up two-thirds of the study sample. About 43% (n = 141) of the study participants were widows/widowers and 33.1% (n = 109) of the study sample were married. The majority of the study participants were primary school leavers (n = 240; 73.0%). The median income for the study sample was 60,000 (range 0-600,000) TZS per month (US$1 ≈ TZS1,300 in 2007). More than three-quarters of the participants (n = 260; 79.6%) earned below TZS 100,000 per month. The majority of the participants (n = 270; 82.1%) were on stavudine, lamivudine and nevirapine combination therapy and the rest (n = 59; 17.9%) were on a zidovudine, lamivudine and efavirenz combination regimen. However, at the time of the study, less than 1% of the recipients had switched between the 2 treatments a couple of times.

**Table 1:** The distribution of the study sample characteristics (*N* = 329)

Characteristics	n(%)
**Sex**	329
Males	112 (34.0)
Females	217 (66.0)
**Age (years)**	329
≤24	7 (2.1)
25-34	67 (20.4)
35-44	154 (46.8)
45-54	77 (23.4)
55-64	20 (6.1)
^3^65	4 (1.2)
Mean (SD)	40.9 (8.7)
**Marital status**	329
Single	20 (6.1)
Married	109 (33.1)
Divorced/separated	59 (17.9)
Widow/widower	141 (42.9)
**Level of education**	329
No formal education	26 (7.9)
Elementary education	240 (73.0)
Post-elementary education	63 (19.2)
^a^**Income/month (TZS)**	327
<100 000	260 (79.6)
100 000-300 000	57 (17.3)
>300 000	10 (3.0)
**Employment**	329
Unemployed	24 (7.3)
Peasants/fishermen/animal keepers	197 (59.9)
Formal employment/business	108 (32.8)
**Religion**	329
Christians	290 (88.1)
Muslims	39 (11.9)

SD, standard deviation; TZS, Tanzanian shillings (US$ 1 ≈ TZS 1,300 in 2007).

a Two participants’ data were missing.

### Self-reported NCDs among the HAART recipients

Self-reported NCDs were common among study participants. Those who reported having at least 1 NCD were 190 out of 329 (57.8%). All the 190 participants who reported having NCDs had either arthritis only or arthritis with some other NCD(s). Thus, arthritis was the commonest NCD. In all the participants, the arthritis was not infective and had existed for at least 3 months. The other NCDs, expressed as a percentage of the whole sample (N = 329), were peptic ulcer disease 38 (11.6%), hypertension 18 (5.5%), bronchial asthma 11 (3.3%), cerebrovascular accidents 9 (2.7%), heart diseases 6 (1.8%), diabetes mellitus 4 (1.2%), epilepsy 2 (0.6%), and cancer 1 (0.3%). The "other" group comprised less frequent conditions that mainly included non-specific chronic symptoms and signs pointing to undiagnosed underlying diseases. As a group, these "other" conditions were 13 (4.0%). The question on NCDs allowed multiple responses. The total responses for all NCDs were 292. As a percentage of the total NCD responses, arthritis accounted for 190 (65.1%), peptic ulcer disease 38 (13.0%), hypertension 18 (6.2%), bronchial asthma 11 (3.8%), cerebrovascular accidents 9 (3.1%), heart diseases 6 (2.1%), diabetes mellitus 4 (1.4%), epilepsy 2 (0.7%) and cancer 1 (0.3%). The "other" group of NCDs accounted for 13 (4.5%).

### Association between socio-demographic characteristics and SF-36 scores of the HAART recipients

Table 2 presents the association between the HAART recipients’ socio-demographic characteristics and their HRQOL. Recipients within the age bracket 35-44 years had a significantly higher mean score for general health perceptions than those in the age group 45-54 years by 8.12 points (p = 0.021). The mean score for role-physical item of the HAART recipients with post-primary education was higher than the mean score of those with primary education only by 18.5 points (p = 0.002). The rest of the socio-demographic characteristics were not significantly associated with HRQOL scores of HAART recipients.

**Table 2:** The relationship between socio-demographic characteristics of HAART recipients and their mean SF-36 scale scores

	Mean scale score (standard deviation)								
	n	PF	RP	BP	GH	VT	SF	RE	MH
**Entire sample**	329	82.4 (20.0)	57.1 (32.6)	73.7 (21.7)	61.5 (20.0)	66.8 (21.8)	73.8 (23.6)	72.1 (23.6)	83.7 (20.0)
**Sex**									
Female	217	79.2 (19.2)	60.7 (39.8)	66.9 (25.0)	64.7 (17.7)	64.6 (19.2)	74.4 (23.6)	53.0 (41.2)	67.5 (22.1)
Male	112	83.2 (18.2)	64.3 (38.1)	66.2 (25.4)	60.9 (20.1)	65.8 (19.0)	75.1 (23.3)	62.2 (40.2)	70.1 (21.2)
*T*-statistics (*p*-value)		1.9 (0.06)	0.8 (0.44)	0.2 (0.82)	1.8 (0.08)	0.65 (0.52)	0.3 (0.78)	1.9 (0.06)	1.0 (0.31)
**Age group (years)**									
≤24	7	84.3 (16.4)	46.4 (39.4)	82.5 (25.7)	55.0 (22.2)	70.0 (18.8)	73.2 (23.3)	61.9 (35.7)	74.9 (16.7)
25-34	67	83.7 (19.6)	65.6 (37.7)	71.0 (24.6)	65.9 (16.4)	65.1 (18.8)	73.1 (25.4)	57.7 (39.3)	68.1 (22.1)
35-44	154	80.6 (18.6)	62.8 (38.5)	65.8 (26.1)	66.2 (17.4)	65.3 (19.9)	75.1 (22.3)	52.4 (41.0)	68.3 (22.3)
45-54	77	79.6 (18.4)	56.9 (42.1)	62.6 (29.0)	58.0 (20.2)	64.6 (20.2)	74.7 (24.6)	61.9 (43.0)	68.1 (22.8)
55-64	20	73.3 (20.1)	63.8 (41.6)	68.9 (21.5)	58.1 (17.4)	60.3 (12.1)	76.3 (17.4)	53.3 (42.5)	67.8 (21.8)
≥65	4	75.0 (15.8)	81.3 (24.0)	63.9 (10.6)	59.0 (8.8)	66.3 (23.6)	75.0 (17.6)	66.7 (47.2)	70.0 (10.6)
*F*- statistics (*p*-value)		1.2 (0.31)	0.8 (0.55)	1.4 (0.24)	3.0 (0.021)^a^	0.4 (0.88)	0.1 (0.99)	0.7 (0.65)	0.1 (0.98)
**Marital status**									
Single	20	84.8 (17.9)	55.0 (46.1)	77.8 (22.8)	61.3 (20.6)	63.3 (19.7)	76.9 (21.5)	55.0 (46.1)	69.8 (28.2)
Married	109	82.9 (16.7)	60.9 (40.7)	67.3 (24.0)	62.6 (17.7)	65.8 (19.8)	77.1 (23.0)	60.9 (56.4)	70.6 (19.8)
Separated	59	79.9 (19.2)	59.9 (41.5)	65.3 (28.4)	64.8 (16.9)	64.0 (18.4)	76.5 (23.0)	51.1 (26.1)	66.5 (23.0)
Widow/widower	141	78.5 (19.0)	51.1 (40.4)	65.1 (26.1)	63.7 (19.0)	64.8 (19.0)	71.6 (23.7)	51.1 (40.4)	67.2 (22.6)
*F*- statistics (*p*-value)		1.6 (0.20)	0.7 (0.55)	1.5 (0.22)	0.3 (0.84)	0.2 (0.92)	1.4 (0.25)	1.4 (0.26)	0.7 (0.57)
**Level of education**									
No formal education	26	80.4 (19.4)	66.3 (39.3)	76.5 (25.5)	61.4 (16.3)	67.9 (16.8)	72.1 (25.0)	52.6 (45.4)	67.7 (20.9)
Primary	240	80.5 (18.6)	57.7 (38.7)	64.9 (24.8)	63.2 (18.6)	64.3 (20.1)	73.3 (23.2)	54.3 (40.3)	67.6 (21.7)
Post-primary	63	81.0 (19.0)	76.2 (34.9)	69.1 (26.2)	64.9 (17.3)	66.1 (18.3)	80.8 (20.6)	64.6 (42.1)	71.6 (21.4)
*F*- statistics (*p*-value)		0.0 (0.98)	5.89 (0.002)^b^	2.8 (0.06)	0.4 (0.68)	0.6 (0.56)	2.7 (0.06)	1.6 (0.20)	0.8 (0.44)
**Income/month (TZS)**									
<100,000	260	80.8 (17.7)	60.4 (38.7)	67.1 (25.8)	63.5 (17.7)	65.4 (19.3)	74.8 (22.6)	55.9 (38.7)	68.3 (22.6)
100,000-300,000	57	79.4 (18.9)	65.8 (37.7)	63.7 (24.2)	62.6 (17.4)	62.8 (16.6)	73.9 (22.6)	53.8 (41.5)	68.1 (21.9)
>300,000	10	81.5 (17.4)	80.0 (35.1)	71.1 (23.1)	65.9 (19.3)	62.5 (19.3)	75.0 (26.2)	76.7 (41.7)	71.2 (19.9)
*F*- statistics (*p*-value)		0.2 (0.86)	1.5 (0.22)	0.6 (0.58)	0.2 (0.86)	0.5 (0.60)	0.0 (0.97)	1.3 (0.27)	0.1 (0.92)

PF, physical functioning; RP, role-physical; BP, bodily pain; GH, general health; VT, vitality; SF, social functioning; RE, role-emotional and MH, mental health.

a By Bonferroni test, GH perceptions mean score of those aged 35-44 was higher than those aged 45-54 by 8.12 points (*p* = 0.021).

b The mean score for the RP scale of those who had post-primary education was higher than that of primary school leavers by 18.5 points (*p* = 0.002). TZS, Tanzanian shillings.

### Impact of NCDs on HRQOL

The PAF for NCDs on physical functioning scale scores was 0.28. The results of the final logistic regression model for physical functioning item score as the outcome variable are presented in Table 3. CD4+ cell count change over time was found to be an interaction factor for the relationship between the NCDs and physical functioning scores of the HAART recipients. In other words, the effect of the NCDs on physical functioning was different in recipients who had an increase in CD4+ cells over time compared with recipients who had no increase in CD4+ cells. Recipients with NCDs and a CD4+ cell increase had a 12.3-fold higher likelihood of having a physical functioning mean score that was below the mean for the general population. On the other hand, in HAART recipients with no CD4+ cell increase, the relationship between the presence of NCDs and the mean physical functioning score was not statistically significant.

**Table 3:** Multiple logistic regression model results for the association between NCDs and physical functioning item score of HAART recipients (*N* = 329)

CD4+ cell status	*n*	aOR for scoring < µ of the general population	95% CI
**CD4+ cell count rise**			
NCDs present	66	12.3	4.3, 22.9
NCDs absent	57	1.0	
**CD4+ cell count decline or same**			
NCDs present	48	3.3	0.4, 5.2
NCDs absent	43	1.0	

The following variables were entered in the global model but turned out not to be significant: sex, age-group, education level, income per month, HAART side effects and adherence to antiretroviral drugs. Missing CD4+ cell count records were 115. NCDs, Non-communicable diseases; HAART, highly active antiretroviral therapy; aOR, adjusted odds ratio; µ, mean; CI, confidence interval.

The PAF for NCDs on mental health scale scores was 0.22. The results of the final logistic regression model with mental health item scale scores as the outcome variable are shown in Table 4. Recipients who had NCDs had a 3.4 times higher risk of scoring less than the general population's mean for the mental health item scale scores (p = 0.005). The relationship between each of the explanatory variables: HAART side effects, adherence to ARVs, CD4+ cell count change, and the outcome variable mental health scores of HAART recipients was not significant.

**Table 4:** Multiple logistic regression model for the association between NCDs and mental health item score of HAART recipients (*N* = 329) adjusted for HAART side effects, adherence to anti-retroviral drugs and CD4+ cell count change

Factors	aOR	95% CI	*P*-value
NCDs	3.4	2.1, 6.5	0.005
Adherence to antiretroviral drugs^a^	1.1	0.9, 5.8	0.123
CD4+ cell count increase/decrease^a^	0.9	0.7, 3.5	0.663
HAART side effects present^a^	1.3	0.5, 6.7	0.312

a Variables forced to remain in the model even though not significant because they have been previously associated with health-related quality of life. The other variables that were not significant were sex, age-group, income per month and education level.

NCDs, Non-communicable diseases; HAART, highly active antiretroviral therapy; aOR, adjusted odds ratio; CI, confidence interval.

## Discussion

### Summary of the findings

This study revealed high prevalence of self-reported NCDs among PLWHA on HAART at the Kagera Regional Hospital in Tanzania. The results also showed that NCDs were associated with the low HRQOL of the HAART recipients. The PAFs for NCDs on physical functioning and mental health scale scores were 0.28 and 0.22, respectively. In other words, 28% of the physical functioning scores and 22% of the mental health scores that were below the mean for the general Tanzanian population could have been improved if all HAART recipients at the Kagera Regional Hospital had no NCDs or had NCDs that were well controlled.

### Findings in the context of previous studies

In a different study conducted at the hospital, it was found that the health transition (a comparison of the current health status to the health status in the previous year) of the HAART recipients was favourable, with more than 88% reporting better health status than in the previous 1 year [4]. The favourable health transition that was found demonstrates the usefulness of HAART despite the negative effects of the NCDs.

Other researchers have also found NCDs to be a risk factor for low HRQOL in HAART recipients. Co-morbidity, in general, has been found to influence HRQOL outcomes in HAART recipients [12, 23, 27, 28]. In the present study, 57.8% of all the study participants reported having at least 1 NCD. It was also found that the effect of the NCDs on physical functioning was different in participants who recorded a CD4+ cell increase compared with those who recorded no increase. In participants who had the CD4+ cell increase; those with NCDs were 12.3 times more likely to have a physical functioning mean score that was less than the score of the general population. In participants who had no CD4+ cell increase, the NCDs did not affect their physical functioning mean score. A possible explanation for this might be that the degree of immunodeficiency in participants who had no increase in the CD4+ cell count was too high to show any significant gain in physical functioning from the absence of the NCDs. It was also found that the HAART recipients who had NCDs had a 3.4-fold higher likelihood of having a mental health score that was below the mean for the general population.

Katusiime and Kambungu [29] studied the prevalence of NCDs among HIV-infected patients in Uganda and found that 27.3% had hypertension and 6.5% had diabetes mellitus. The percentages are higher than what was found in this study, which may be explained by the fact that the HIV-infected patients studied in Uganda were all aged 60 years and above. Chu et al [7] found that the prevalence of hypertension and diabetes mellitus in HIV-infected adults in Bronx New York ranged from 13 to 48%. The self-reported prevalence of hypertension (5.5%) and diabetes mellitus (1.2%) in the present study were lower than those found by Chu et al. These differences may be explained by the differences in the prevalence of NCDs in the general population where the studies were conducted.

### Limitations and future research

This study had some limitations. The SF-36 health survey does not measure quality of life in totality and is not HIV-specific. Comparison of the prevalence of NCDs in the study population to that in the general population would have been very informative. However, the prevalence of NCDs in the general population in study area has not been described. Further, the cross-sectional design employed does not provide evidence of the direction of association because it fails to distinguish which preceded the other between the explanatory and outcome variables. Future research should use HIV-specific HRQOL measurements. However, these need to be validated first before use on the target population. Whenever resources allow, a longitudinal approach should be employed.

## Conclusion

Self-reported NCDs were prevalent among HAART recipients in Kagera and were associated with low HRQOL in the HAART recipients. This finding has important health care implications. It calls for a holistic and collaborative approach to the care and treatment of PLWHA as NCDs varied widely and required expertise cutting across many different specialties. The control of the NCDs would considerably improve the HRQOL of the HAART recipients. Health care workers who attend to PLWHA at the CTCs should be trained to diagnose and treat a wide range of conditions like arthritis, peptic ulcer disease, hypertension, bronchial asthma, cerebrovascular disease, heart disease and diabetes mellitus. Refresher courses that cover the management of the common NCDs that are encountered in the HIV/AIDS field are recommended.
